# Predicting distribution of malaria vector larval habitats in Ethiopia by integrating distributed hydrologic modeling with remotely sensed data

**DOI:** 10.1038/s41598-021-89576-8

**Published:** 2021-05-12

**Authors:** Ai-Ling Jiang, Ming-Chieh Lee, Guofa Zhou, Daibin Zhong, Dawit Hawaria, Solomon Kibret, Delenasaw Yewhalaw, Brett F. Sanders, Guiyun Yan, Kuolin Hsu

**Affiliations:** 1 Department of Civil and Environmental Engineering, University of California Irvine, Irvine, CA USA; 2 Program in Public Health, University of California Irvine, Irvine, CA USA; 3 Yirgalem Hospital Medical College, Yirgalem, Ethiopia; 4 Tropical and Infectious Diseases Research Center (TIDRC), Jimma University, Jimma, Ethiopia; 5 Department of Medical Laboratory Sciences, Institute of Health, Jimma University, Jimma, Ethiopia; 6 Department of Urban Planning and Public Policy, University of California Irvine, Irvine, CA USA

**Keywords:** Malaria, Ecological modelling, Hydrology

## Abstract

Larval source management has gained renewed interest as a malaria control strategy in Africa but the widespread and transient nature of larval breeding sites poses a challenge to its implementation. To address this problem, we propose combining an integrated high resolution (50 m) distributed hydrological model and remotely sensed data to simulate potential malaria vector aquatic habitats. The novelty of our approach lies in its consideration of irrigation practices and its ability to resolve complex ponding processes that contribute to potential larval habitats. The simulation was performed for the year of 2018 using ParFlow-Common Land Model (CLM) in a sugarcane plantation in the Oromia region, Ethiopia to examine the effects of rainfall and irrigation. The model was calibrated using field observations of larval habitats to successfully predict ponding at all surveyed locations from the validation dataset. Results show that without irrigation, at least half of the area inside the farms had a 40% probability of potential larval habitat occurrence. With irrigation, the probability increased to 56%. Irrigation dampened the seasonality of the potential larval habitats such that the peak larval habitat occurrence window during the rainy season was extended into the dry season. Furthermore, the stability of the habitats was prolonged, with a significant shift from semi-permanent to permanent habitats. Our study provides a hydrological perspective on the impact of environmental modification on malaria vector ecology, which can potentially inform malaria control strategies through better water management.

## Introduction

Long-lasting insecticide-treated nets (LLINs) and indoor residual spray (IRS) are the key tools for malaria vector control^1,2^. Scale-up of LLINs and IRS in the past decade has reduced malaria burden in Africa by half^3^, however the progress of malaria control has stalled in many African countries due to limited efficacy of LLINs and IRS as a result of insecticide resistance and increased outdoor biting behavior^4^. Thus, there is a recent renewed interest in larval source management (LSM) as a supplementary vector control tool^5^. LSM involves larviciding and biological control of malaria vectors, and also modification and manipulation of aquatic habitats^5^. LSM has not been widely used in malaria vector control in Africa, partly due to the challenge of widespread and unstable larval sites in many ecosystems. LSM may not be suited to all ecosystems, however LSM would be greatly facilitated if larval habitat distribution under natural climatic conditions can be predicted a priori, so that regions best suited to LSM can be identified. Further, prediction of how environmental modification such as irrigation, canal construction and landscape alteration through engineering approach may change the distribution of transient, semi-permanent and permanent aquatic habitats would greatly help LSM-based malaria vector control program which is much needed in Africa.

The LSM program requires identification of aquatic habitats for malaria vectors. Past studies have attempted to use field-based surveys or harness remotely sensed data for larval habitat identification^6,7^. Field-based surveys involve the use of manual labor or unmanned aerial vehicles (UAVs) with geographic information system (GIS) to map larval habitats, which can be time consuming^4^, limited in geographic coverage and weather dependent^8,9^. Alternatively, researchers have relied on satellite imagery and supervised classification into land use and land cover (LULC) maps to delineate potential larval aquatic habitats^10–12^. The type of satellite imagery used is usually optical, which tends to be limited by cloud cover and is unable to identify water bodies hidden by vegetation cover^13,14^.

An alternative approach is required as the aforementioned inadequacies in the existing methods often result in larval habitat mapping of limited coverage or discontinuous frequencies that are unable to support effective LSM. We propose a novel approach using a physics-based integrated hydrological model that draws on fundamental principles to realistically model potential larval aquatic habitats. Complex hydrologic processes built into the model such as infiltration, evapotranspiration and runoff help to provide a mechanistic understanding of aquatic habitat behavior. The primary inputs of a hydrological model, namely meteorological and topographic datasets, can be acquired globally and are available at high temporal and spatial resolutions respectively through remote sensing^15,16^. Notably, the larvae of the major malaria vector in Ethiopia, *Anopheles arabiensis*^17^, have been associated with transient pools^18^ and our approach allows the larval habitats to be resolved down to sub-daily frequencies and tens of meters resolutions necessary to capture the dynamic nature of the habitats. It can also be scaled up in coverage if required.

Several studies in malaria transmission have incorporated hydrologic modeling. Soti et al*.* combined a simple water balance model with a mosquito population model to predict the abundance of mosquitoes contributing to the transmission of Rift Valley fever in West Africa^19^. Asare et al*.* applied another simplified water balance model (VECTRI) and parameterized the processes to simulate the fractional water coverage in central Ghana^20^. The empirical nature of the model and unrealistic assumptions made about infiltration and runoff result in heavy reliance on calibration and can increase model uncertainty substantially. Bomblies et al. used a mechanistic hydrologic model to simulate the surface water area for two villages in Niger^21^. However, the subsurface and surface water components are only coupled one-way such that surface water can only flow to the subsurface but not the other way round. The lack of exfiltration and re-infiltration components precludes the representation of spring-fed pools from groundwater recharge. Additionally, the model did not account for lateral subsurface flow, which can influence evapotranspiration and redistribute groundwater to low-lying areas, especially at higher spatial resolutions^22,23^. To simulate the dynamics of the aquatic larval habitats, the hydrologic model chosen must be able to detail the surface–subsurface interactions and plant processes associated with ponding given the complex interdependence between larval habitats and the environment. In addition, none of the existing hydrology-based malaria models have been used to investigate the impact of irrigation on larval habitats.

In the present study, we aim to examine the potential of integrated hydrological models in predicting the location of mosquito larval habitats by capturing the shallow subsurface dynamics and improve our understanding of the hydrologic processes and environmental modifications that render larval habitats. Specifically, we seek to answer the following: (1) Where are the potential larval habitats located and what is the probability of occurrence? (2) How long can the larval habitats be sustained? (3) Is there a cyclical pattern in the extent of the larval habitats? (4) What is the impact of irrigation on each of the above? The uniqueness of our approach lies in its consideration of irrigation practices and its ability to resolve complex ponding processes that contribute to potential larval habitats such as groundwater-surface water interactions^24^. We chose ParFlow^22,25–28^ for its open-source nature, robust numerical solver^28^, and compatibility with high-performance computing^29^. To take into account irrigation and land cover characteristics, ParFlow was coupled with the Community Land Model (CLM)^30^ to simulate soil moisture for the identification of malaria larval habitats in a sugarcane plantation and its vicinity in Arjo, Ethiopia.

## Methods

### Model description

ParFlow has been applied in many studies to simulate complex surface–subsurface interactions in heterogeneous environments^31,32^. Richards’ equation^33^, which governs water movement through the unsaturated zone, is used to simulate subsurface flow in three dimensions. The diffusive wave and Manning's equations, which calculate the depth and velocity of the routed water, are used to represent the overland flow in two dimensions^22^. To connect the overland flow and subsurface, the former is imposed as a boundary condition on the latter for natural feedbacks between the two components^22^.

Considering the non-linear nature of the governing equations, ParFlow solves the coupled system implicitly using the Newton–Krylov method for robust convergence to the solution and multigrid preconditioning for parallel scalability. This allows the system to be solved efficiently through parallel computing^29^. For details, see Ashby and Falgout^25^, Jones and Woodward^26^, and Kollet and Maxwell^22^. Additionally, CLM simulates the land surface water and energy balance which includes evaporation, transpiration, snow processes, heat fluxes, and radiation partitioning^30,34^. The water fluxes calculated by CLM are incorporated in ParFlow through the source or sink terms in Richards’ equation for subsurface flow^30^. The two models are coupled over a user-defined number of subsurface layers and this allows ParFlow to take into account the characteristics of the vegetation cover as CLM simulates plant function types corresponding to different vegetation parameters^27,30^.

### Study area

**Figure 1:**
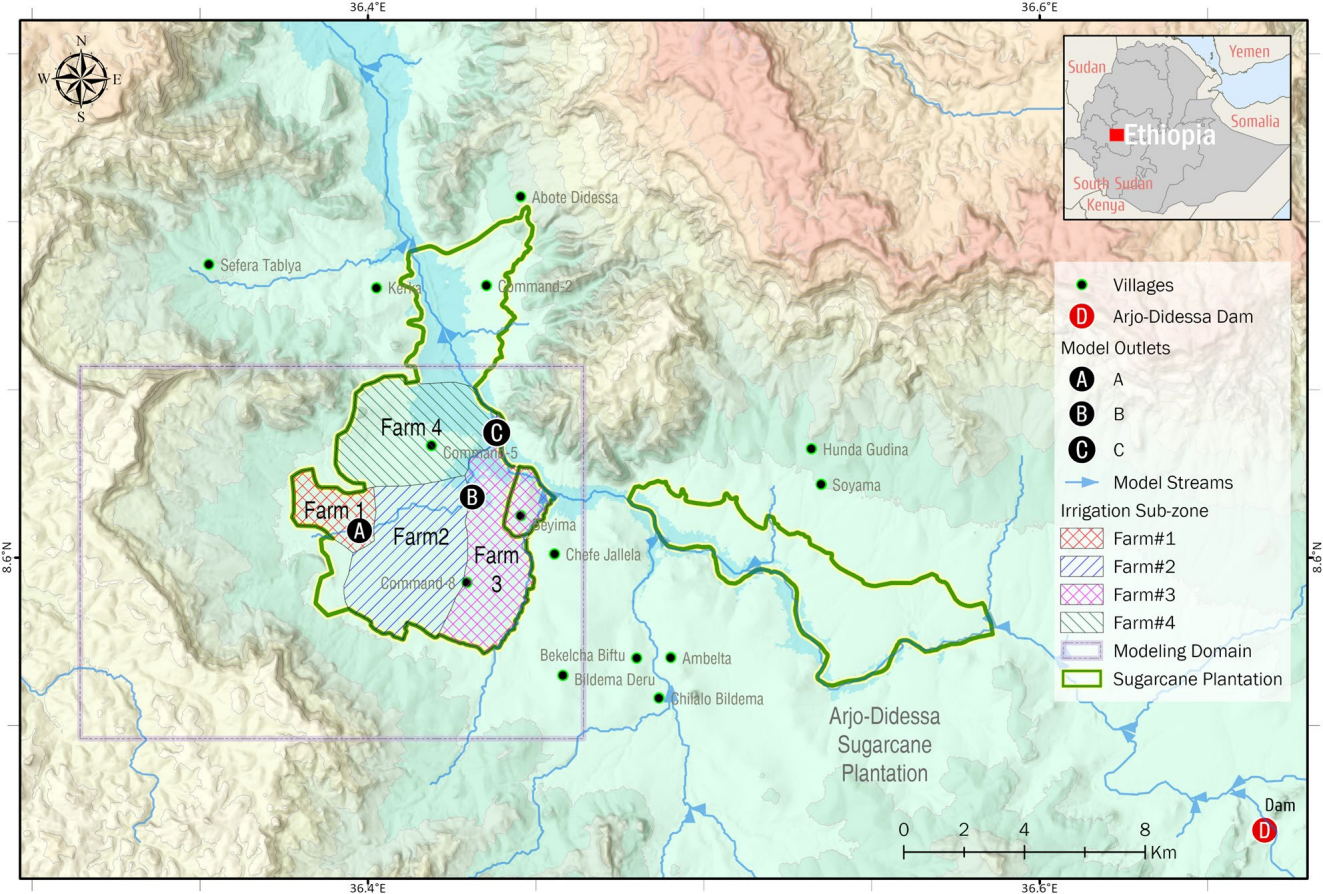
Study area at Arjo-Dedissa sugarcane plantation and its vicinity. This area is found in the Oromia Region of western Ethiopia and located 395 km west of the capital, Addis Ababa, at the intersection of the three woredas (districts), Jimma Arjo (East Wollega Zone), Bedele (Buno Badale Zone), and Dabo Hana (Illubabor Zone) at the Didessa River valley. The model area is enclosed by the gray box. The sugarcane plantations in the study area were demarcated by the green lines. To simplify model simulation, the irrigation parcels in the plantation area were further grouped and generalized into four farms, which will be explained in greater detail in the later subsection.

The study area is 208 km^2^ and comprises Arjo-Didessa sugarcane plantation and its vicinity in the Oromia Region State, western Ethiopia (Fig. 1). The altitude of the study area is 1,350 m above sea level and the annual rainfall received is 1,477 mm^35^, with a rainy season between May and October. The area covers most of the Arjo-Didessa sugarcane plantation site, which is characterized by clay and clay loam with low permeability^36^. Due to the slow rate of infiltration, rainwater can accumulate easily and form ponds in the area, which is exacerbated by irrigation of the sugarcane plantation. The widespread and persistent nature of this ponding contributes to the breeding of malaria vector mosquitoes.

A recent study indicated that malaria is seasonal in this area; transmission mainly followed the rainy season, with the highest cases recorded between September and November^37^. However, some localities experienced persistent malaria due to environmental modifications such as irrigation that support the continuous availability of breeding sites. *Anopheles arabiensis* is the predominant malaria vector species in the area. The major mosquito breeding habitat types included rain pools, stream shoreline, animal foot prints, irrigation canal, hippo trenches, drainage ditches, and puddles in rice cultivation^35^ (Supplementary Fig S1).

In the ParFlow-CLM model, the study area was discretized with a resolution of 50 m, resulting in a grid configuration of 332 by 248 cells. The subsurface component was divided into 10 layers and the thickness of each layer varies, depending on the granularity of the data available. In general, the resolution of the subsurface layer increased nearer to the surface to capture the shallow surface processes in greater detail. The layer thicknesses ranged from 0.25 m to 20 m, over a total vertical depth of 100 m.

### Input data

As the model domain is a rural area where field data for model construction was scarce, remotely sensed data and global synthetic datasets from published works were used. For example, 1 arc-second digital elevation model (DEM) from SRTM^38^ was resampled to the 50 m model grid and converted to ground surface slopes as an input to ParFlow. The land cover for each grid cell in CLM was determined by the classification of 30 m resolution Landsat-8 imagery^39^ taken on a cloud-free day in January 2018 into International Geosphere-Biosphere Programme (IGBP) types. To characterize the subsurface, the soil taxonomy distribution (Supplementary Fig S2) for the top 2 m from the surface was referenced from the SoilGrids250m TAXOUSDA dataset^40^. The saturated hydraulic conductivity of the deeper zone beyond the top 2 m was based on GLHYMPS 2.0^41^. The depth to bedrock data from SoilGrids250m BDRICM dataset^40^ was used to delineate the bedrock zone, which was assigned a very low hydraulic conductivity. For the meteorological forcing, 0.04 degree by 0.04-degree precipitation data from Precipitation Estimation from Remotely Sensed Information using Artificial Neural Networks-Cloud Classification System (PERSIANN-CCS)^42,43^ was resampled to the model grid using bilinear interpolation. In addition, wind speed from the second version of Modern-Era Retrospective analysis for Research and Applications (MERRA-2)^44^ of 0.5 degrees by 0.625 degrees resolution and air temperature, pressure, specific humidity and radiation data from Global Land Data Assimilation System (GLDAS)^45^ of 0.25 degree by 0.25 degree resolution were averaged for the entire domain. All the forcing data were obtained from 2018 and input to the model hourly. The list of model input data and the relevant details can be found in Supplementary Table S1 and Supplementary Note Section 1.

### Model scenarios

A 1-year baseline period in 2018 from January 1 to December 31 was simulated with an hourly time step to produce daily soil saturation and groundwater pressure head. Sugarcane is a plant with high water consumption so irrigation during the dry season is essential. Hence, a separate scenario was run for the same period with the implementation of a synthesized irrigation scheme corresponding to the dry season from January to April and November to December. This synthesis was based on the sugarcane plantation irrigation schedule and detailed plans acquired from Arjo-Didessa Sugar Factory in Supplementary Fig S3^36^. Specifically, the sugarcane plantation in the study area was grouped into four irrigation sub-zones as a simplified representation (Fig. 1). In each irrigation cycle, Farms 1 and 3 are sprinkler-irrigated for 10 days, followed by Farms 2 and 4. Each farm receives a total of 10 mm of irrigation over 22 h each day during its turn for irrigation.

**Figure 2:**
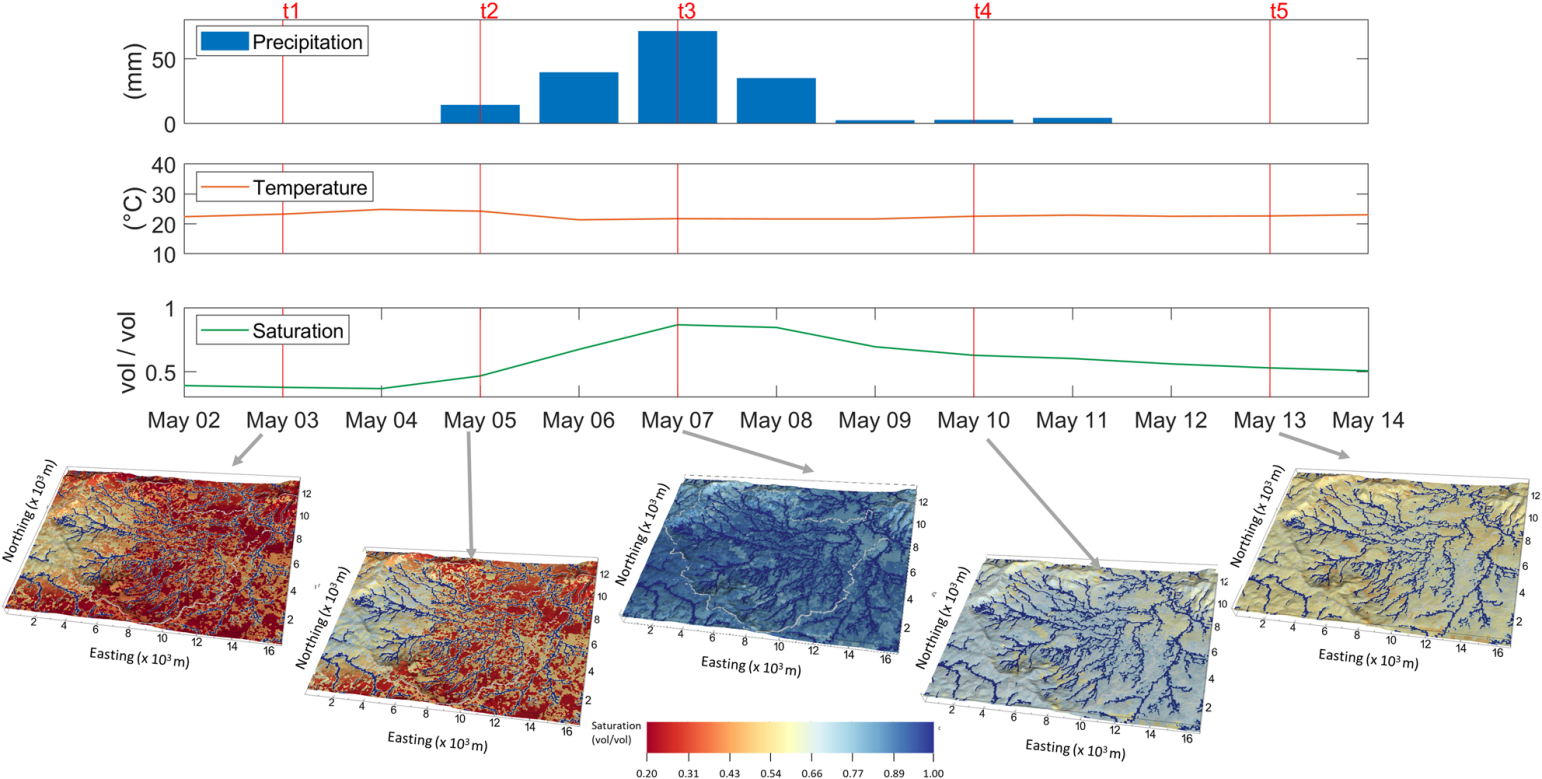
The simulated surface layer (25 cm depth) soil saturation at five time instances during a rain event in May 2018. The snapshots illustrate the close-up views of the soil saturation for the rainfall event between May 5, 2018, and May 11, 2018, along with the time series of the spatially averaged precipitation, temperature, and simulated surface layer. On May 3(t1), the surface was generally dry before the onset of the rainfall, except for the mountainous areas on the left. On May 5 (t2), the rain started to spread from the mountainous areas. By May 7 (t3), the rain had spread to the entire area. The snapshot at t4 shows the post-rainfall distribution on May 10, and the snapshot at t5 shows the area drying up again after the rainfall event on May 13. The soil saturation increased to more than 85% at the peak of the storm across most of the study area and decreased quickly after about 5 days but the streams and the vicinities remain wet. There were no large depressions (e.g. lakes, pools) observed in the simulation.

As an illustration of the hydrological process for the baseline scenario, Fig. 2 shows a time series of the simulated spatially averaged surface layer soil saturation, along with snapshots of the resulting surface layer soil saturation at five particular time instances in May 2018 when a 7-day rainfall event occurred. The instantaneous snapshots of the soil saturation reflect the spatial distribution of the rainfall. The details of the baseline simulation for the entire year and the irrigation scenario results can be found in Supplementary Fig S4, Supplementary Fig S5, and Supplementary Note Section 2. The animations of the simulated soil saturation dynamics for the baseline condition and irrigation scenario with daily precipitation in 2018 can be found in Supplementary Videos S1 and S2.

### Wetness index calculation

As mosquito reproduction is successful only if larval habitats remain stable for a period sufficient to sustain the aquatic stage^46,47^, the viability of the habitat was determined by the persistence of ponding. Hence, we developed a Wetness Index (*WI*) metric to quantify the persistence of ponding as a basis for potential habitat representation after rain and irrigation. This will be used later on to answer our research questions.

As the aquatic habitats at site were typically shallow and the lateral scale was of the order 10 m or smaller, it was not feasible to explicitly simulate the surface water depth of the individual habitat. Hence, the simulated soil saturation of the top surface layer as described in the previous section, coupled with a threshold, was used to assess the availability of the surface water that could contribute to ponding. Since soil saturation measures the extent to which the water content has filled up the voids within the soil, a higher soil saturation means there is a larger volume of water stored in the soil within the 50 m grid cell. Hence, a potential occurrence of ponding was assumed if the surface layer soil saturation exceeded the threshold. Otherwise, no ponding occured. To evaluate the duration of ponding, *WI* was used and defined as the cumulative number of days of ponding from the start of the simulation year at any grid cell (*x,y*) and day (*t*), based on the simulated soil saturation of the top surface layer *S*(*x,y,t*) and a soil saturation threshold *θ*. The computation of the index is as shown below in Eq. (1):

1 $$WI\left( {x,y,t} \right) = \left\{ {\begin{array}{*{20}c} {WI\left( {x,y,t - 1} \right) + 1,\, if\, S\left( {x,y,t} \right) \ge \theta } \\ {0, otherwise} \\ \end{array} } \right.$$

The initial *WI* for every grid cell was set to 0. The index increases each day if the soil saturation exceeds the threshold. Otherwise, it will reset to zero, implying that the habitats in the grid cell are no longer able to sustain the development of the larvae population.

**Figure 3:**
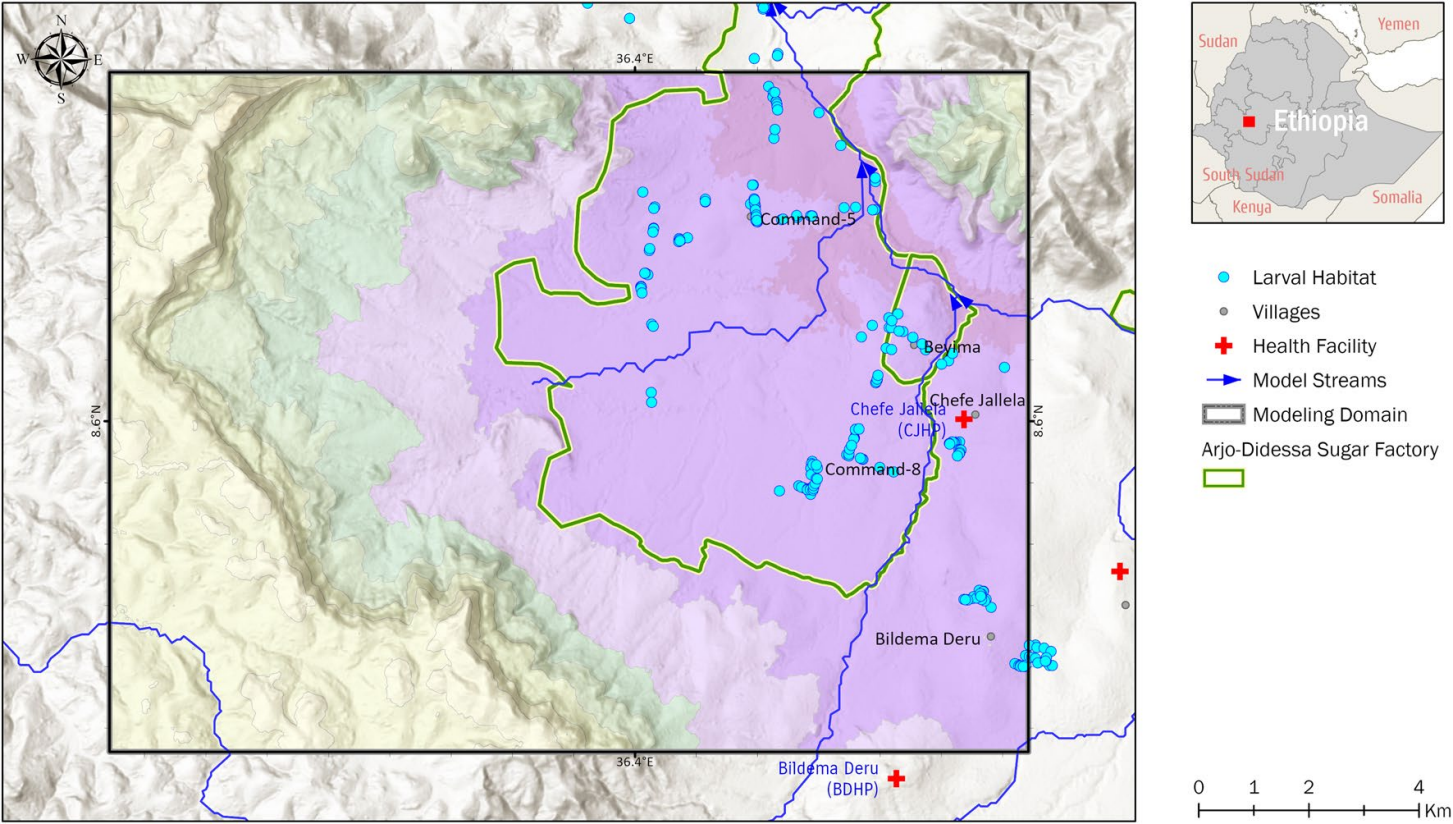
Location of the surveyed aquatic habitats. All accessible potential mosquito breeding habitats were surveyed and identified the presence of mosquito larvae during the dry (December 2017–February 2018) and rainy (June 2018–August 2018) seasons.

The soil saturation threshold *θ* in Eq. (1) was calibrated based on a field survey of aquatic larval habitats. 134 ponding locations were surveyed for larval growth during the dry (December 2017–February 2018) and rainy (June 2018–September 2018) seasons^35^. For each surveyed location, information regarding whether larval growth was detected, the type of species identified, larval density, habitat dimension, habitat type and land use type were recorded. Regardless of whether larval growth was detected, each survey location served as an indication of ponding for calibration and validation. Some of the surveyed locations such as man-made ponds, tire track puddles, and animal footprints which could not be simulated by the hydrologic model were omitted. In addition, to minimize the influence of dry season irrigation on the parameterization considering that the irrigation in the model was approximated by a simplified scheme, the calibration was only conducted for the rainy season from May to October. In total, 102 of the surveyed locations were used for calibration and validation as shown in Fig. 3.

The objective of the calibration was to maximize the probability of detection (*POD*), which determines if the model can predict an aquatic habitat successfully. Other measures which can capture overprediction were not chosen as the field data only covered locations with ponding and it was challenging to rule out small puddles within the grid cell using other types of data. To ensure the relevance of the calibrated *θ*, a bootstrapping method was applied and it was found that the optimal *θ* was 0.48. In other words, the model would predict the occurrence of ponding for soil saturation above 0.48 at locations in line with the survey. Details of the survey data and calibration method can be found in Supplementary Note Section 3.

**Figure 4:**
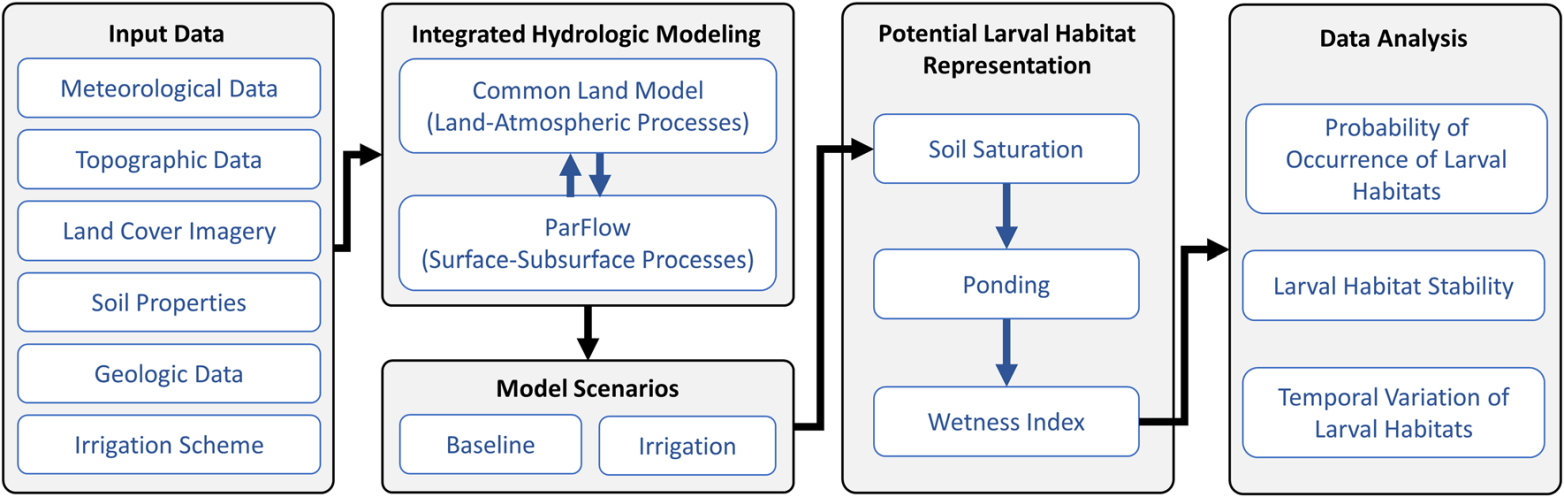
Overall schematic of methodology.

In summary, the overall schematic of our methodology is shown in Fig. 4. Using the Wetness Index, we analyzed the potential larval habitats in terms of their spatial distribution, stability and temporal pattern and the results are presented in the next section.

## Results

### Location of potential larval habitats and probability of occurrence

Generally, *Anopheles arabiensis* mosquito takes around 15 days to develop from egg to adult, but the duration can be as short as 10 days due to selection pressures from the stressed environment such as drought, temperature anomaly, or limited food resources^48,49^. In this regard, we considered areas with *WI* exceeding 10 and 15 days to be potential larval habitats under critical and normal conditions, respectively.

To determine the probability of potential larval habitat occurrence, we computed the probability of ponding occurring longer than 10 and 15 days, *P*(*WI* > *T*)*,* as shown in Eq. (2). *P*(*WI* > *T*) is defined as the ratio of *D*(*WI*(*x,y,t*) > *T*), the number of cumulated days for which the *WI* (i.e. persistence of ponding) of a grid cell (*x*,*y*) at time t that exceeded *T* days, to *D*_*period*_, the number of days within a defined period of simulation.

2 $$P\left( {WI > T} \right) = \frac{{D(WI\left( {x,y,t} \right) > T)}}{{D_{period} }},\,T \in \left\{ {10,15} \right\}$$

**Figure 5:**
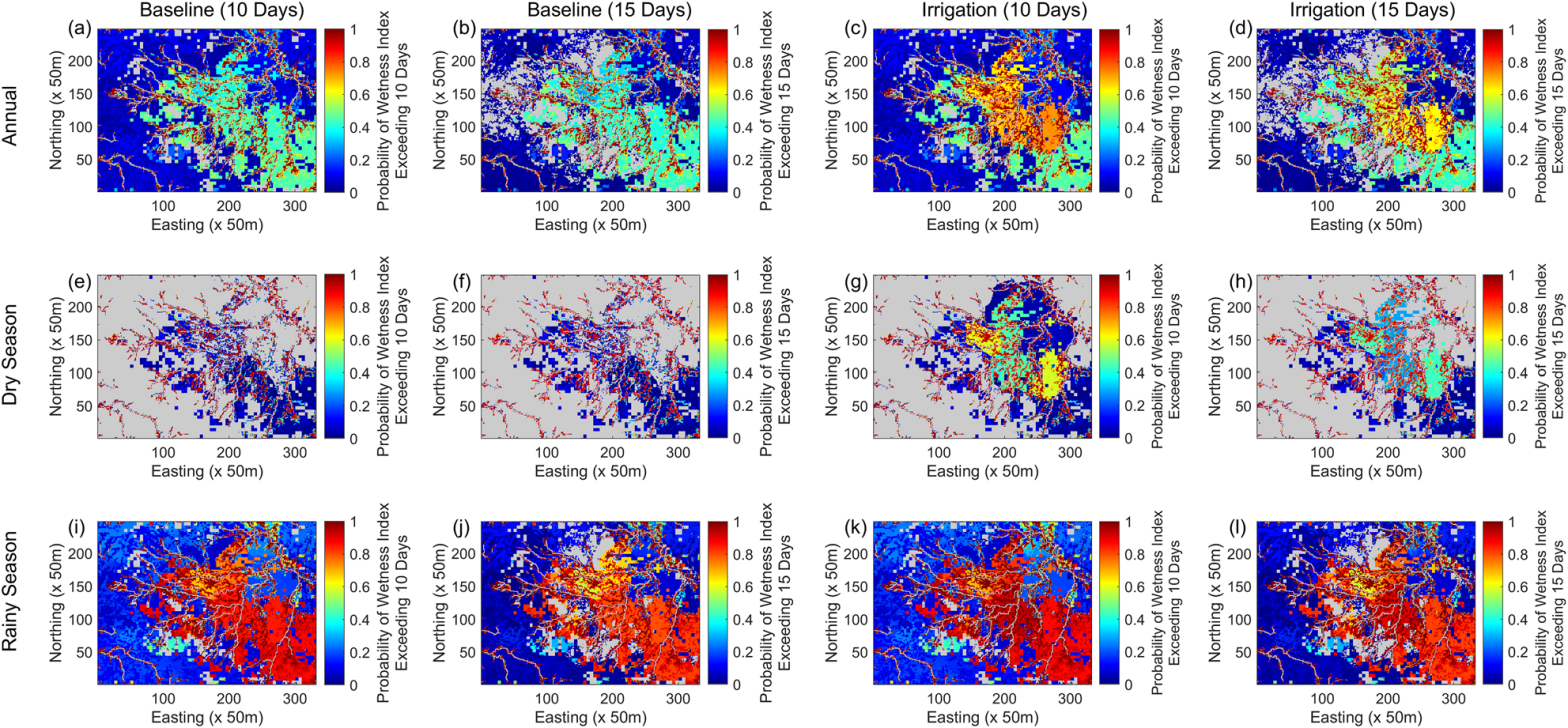
Spatial distribution for the probability of potential larval habitat occurrence. (**a**–**d**) represent the probability of *WI* exceeding 10 days and 15 days for the baseline scenario and the irrigation scenario for the entire year. Similarly, (**e**–**h**) represent the probability of *WI* exceeding 10 days and 15 days during the dry season, and (**i**–**l**) represent the probability of *WI* exceeding 10 days and 15 days during the rainy season. Areas where the simulated surface water flowrate exceeded 0.01 m^3^/s for 90% of the time in the simulated year were masked out for all the sub-figures since *Anopheles* larvae have a lower chance of surviving in fast-moving water^61^.

Figure 5 shows the results for the spatial distribution of *P*(*WI* > *T*) over the three periods of simulation, namely the entire year of 2018, the dry season (i.e. January to April and November to December) and the rainy season (i.e. May to October). It can be observed that ponding was persistent throughout the year around the stream edges and the vicinity. *P*(*WI* > 10) and *P*(*WI* > 15) were consistently close to 1, reflecting a high potential of these areas as larval habitats.

For the baseline scenario shown in Fig. 5a,b, the *P*(*WI* > *T*) for the areas outside of the streams was predominantly determined by soil type. The areas characterized by Usterts (see Supplementary Fig S2) with the lowest hydraulic conductivity in the model domain were the next most at risk, with a *P*(*WI* > *T*) of about 0.4–0.5. In the remaining areas, *P*(*WI* > *T*) was generally 0.2 or less. Comparing Fig. 5a,b, the differences were minimal except for the steep areas at the watershed upstream boundary where *P*(*WI* > 15) was predominantly zero. The surface water ponding was unable to last more than 15 days due to the terrain gradient.

Figure 5c,d show the results for the irrigation scenario. Compared to the baseline scenario, the year-round persistent ponding around the streams and the vicinity was wider in coverage and more noticeable. Irrigation also increased *P*(*WI* > 10) in Fig. 5c and P(*WI* > 15) in Fig. 5d from 0.4–0.5 to about 0.7 and 0.6 respectively for Farm #1, Farm #2, and a significant portion of Farm #3 and Farm #4. The *P*(*WI* > *T*) for the remaining area within the farms remained relatively unchanged at 0.2 and this could be attributed to the Ustoll soil type which drains easily. The increase in the probability of potential larval habitat occurrence from the baseline was more pronounced for *P*(*WI* > 10) than *P*(*WI* > 15) since the interval of irrigation was set at 10 days, after which the farm drained without replenishment until the next irrigation cycle.

For the dry season, it can be observed in Fig. 5e,f that the stream edges were the only areas with high potential of larval habitat occurrence. In Fig. 5g,h, P(*WI* > *T*) increased visibly in the farms after irrigation, with a distinct similarity between Farms #1/#3 and Farms #2/#4 that points to the irrigation schedule. While irrigation was alternated evenly between the two groups, Farms#1 and #3 showed a higher *P*(*WI* > *T*) than Farms #2 and #4, possibly due to the timing of the irrigation relative to the rainfall. Irrigation could either coincide with rainfall or act as a supplement when there was no rainfall to augment soil moisture. Noticeably, there was an area to the northeast straddling both Farm #3 and Farm #4 where *P*(*WI* > 10) was around 0.1 but *P*(*WI* > 15) was almost zero, indicating that irrigation only allowed for larval habitats under critical conditions in that area during the dry season.

For the rainy season, it can be observed in the baseline scenario (Fig. 5i,j) that the areas characterized by Ustert exhibited a high potential of larval habitat occurrence, apart from the stream edges. Particularly, there was an area to the north where *P*(*WI* > *T*) was lower than the other parts which could be due to the relatively steeper terrain. In the irrigation scenario (Fig. 5k,l), there was no visible difference in *P*(*WI* > *T*) as compared to the baseline scenario, apart from a minor increase around the western part of Farm #4.

**Figure 6:**
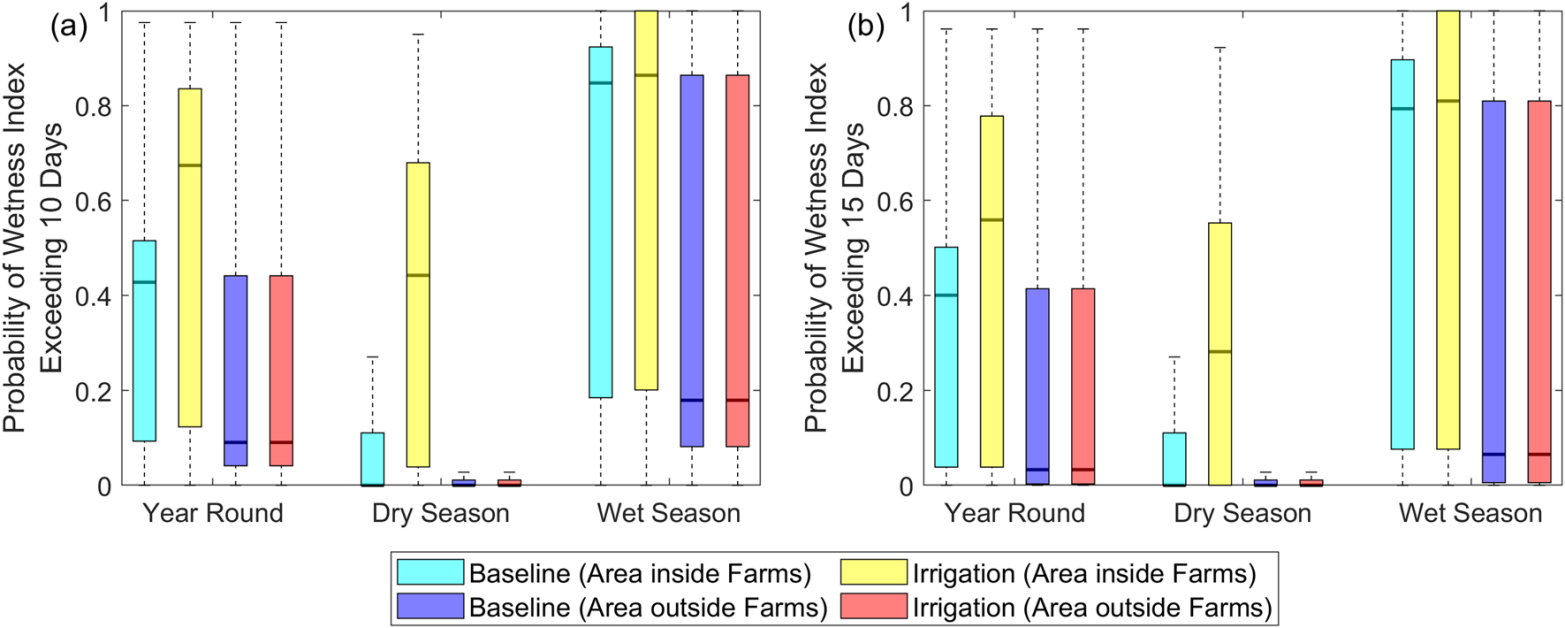
Box plots for the probability of potential larval habitat occurrence for the whole year, dry, and rainy season. Probability of *WI* exceeding (**a**) 10 days and 15 days (**b**) for the area inside farms and the area outside farms. The line within each box is the sample median and the top and bottom of each box are the 25th and 75th percentiles. The whiskers were drawn from the two ends of the box and demarcate the observations which were within 1.5 times the interquartile range from the top and bottom of the box.

As a summary, we present the results in boxplots as shown in Fig. 6 to illustrate the effect of irrigation in different seasons for the areas inside and outside farms. The relevant statistics can be found in Table 1. The *P*(*WI* > *T*) had a highly asymmetrical distribution because it was very low in most of the model domain but could be very high in the remaining areas due to the streams. For the following comparison, we will use the median as it was more representative of the distribution.

**Table 1:** Summary statistics of the probability of potential larval habitat occurence for the whole year, dry season, and rainy season. Mean, 25th percentile (P25), median and 75th percentile (P75) of the probability of *WI* exceeding 10 days and 15 days for the (a) areas inside farms and (b) areas outside farms. The *p* value was derived from the Wilcoxon Rank-Sum test under the null hypothesis that irrigation did not increase the median probability of exceedance from the baseline scenario.

	Baseline				Irrigation				*p* value
	Mean	P25	Median	P75	Mean	P25	Median	P75	
**(a) Area inside Farms**									
*Wetness Index Exceeding 10 Days*									
Dry season	0.173	0.000	0.000	0.111	0.424	0.039	0.442	0.680	< 0.01
Rainy season	0.607	0.185	0.848	0.924	0.643	0.201	0.864	1.000	< 0.01
Year round	0.392	0.093	0.427	0.515	0.534	0.123	0.674	0.836	< 0.01
*Wetness Index Exceeding 15 Days*									
Dry season	0.168	0.000	0.000	0.111	0.347	0.000	0.282	0.553	< 0.01
Rainy season	0.553	0.076	0.794	0.897	0.597	0.076	0.810	1.000	< 0.01
Year round	0.362	0.038	0.400	0.501	0.473	0.038	0.559	0.778	< 0.01
**(b) Area outside farms**									
*Wetness Index Exceeding 10 Days*									
Dry season	0.053	0.000	0.000	0.000	0.053	0.000	0.000	0.000	0.254
Rainy season	0.202	0.082	0.120	0.201	0.202	0.082	0.120	0.201	0.437
Year round	0.128	0.041	0.060	0.101	0.128	0.041	0.060	0.101	0.430
*Wetness Index Exceeding 15 Days*									
Dry season	0.051	0.000	0.000	0.000	0.051	0.000	0.000	0.000	0.385
Rainy season	0.125	0.005	0.038	0.098	0.125	0.005	0.038	0.098	0.440
Year round	0.089	0.003	0.019	0.049	0.088	0.003	0.019	0.049	0.443

In the baseline scenario, there was a higher potential for larval habitats to form inside the farms, with a median *P*(*WI* > 10) of 0.427 and a median *P*(*WI* > 15) of 0.400, than outside the farms, with a median *P*(*WI* > 10) of 0.06 and a median *P*(*WI* > 15) of 0.019. This is expected because the farms are located in an area with relatively flat terrain and a higher concentration of streams. The difference in the median *P*(*WI* > *T*) inside and outside the farms was bigger in the rainy season compared to the dry season, as the higher rainfall received intensified ponding.

Irrigation increased the median *P*(*WI* > *T*) inside the farms drastically in the dry season, with the median *P*(*WI* > 10) increasing from 0 to 0.442 and the median *P*(*WI* > 15) increasing from 0 to 0.282. Although irrigation was only applied over the dry season, there was also a statistically significant increase in the median *P*(*WI* > *T*) during the rainy season (*p* < 0.01). The median *P*(*WI* > 10) increased from 0.848 to 0.864 while the median *P*(*WI* > 15) increased from 0.794 to 0.810. This was due to irrigation contributing to the antecedent soil moisture before the onset of the rainy season, which shortened the time for the soil to become saturated and ponding to occur. On the other hand, there was no strong evidence outside the farms of an increase in the median *P*(*WI* > *T*) due to irrigation (*p* > 0.01). This applied to both rainy and dry seasons.

### Stability of larval habitats

**Figure 7:**
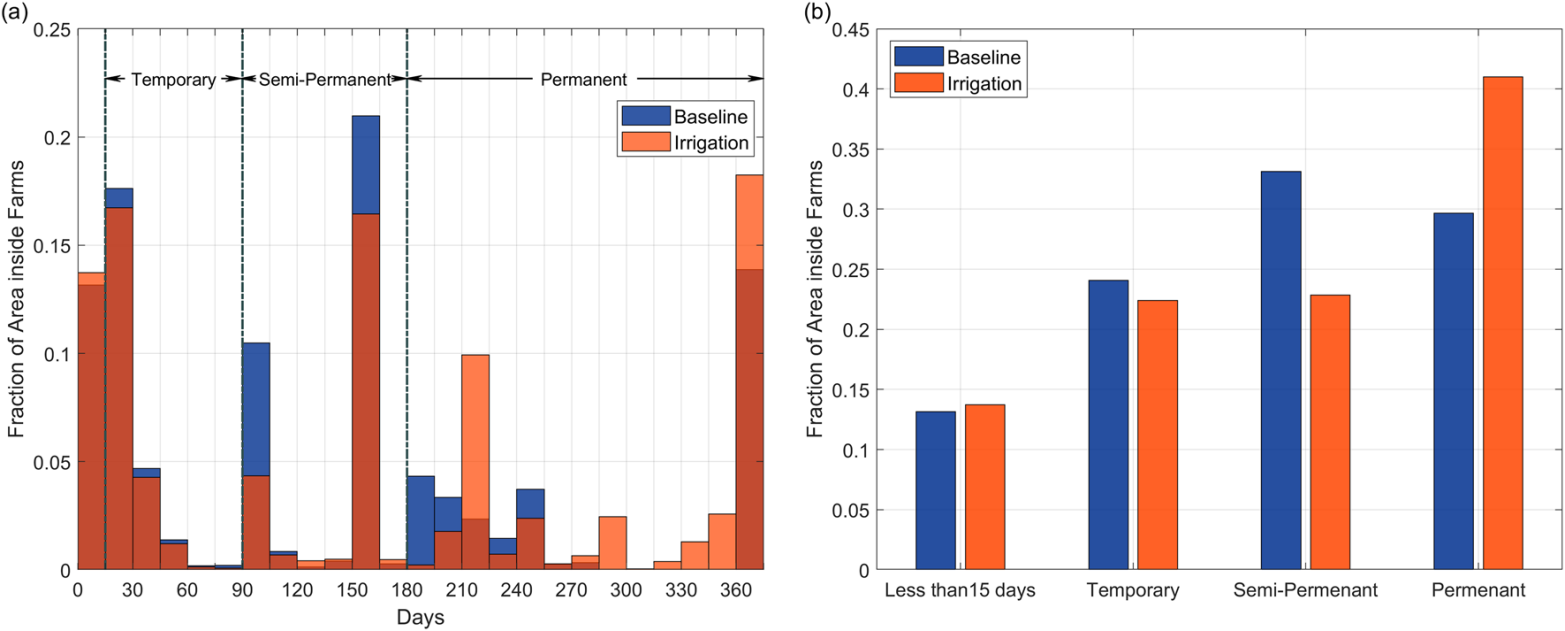
The fraction of area inside the irrigated farms for each potential larval habitat types under the baseline and irrigation scenarios. (**a**) Shows the histogram of the maximum duration of ponding within the year for the grid cells in each type of habitats expressed as a fraction of the total area of the farms. The bin size was 15 days. Temporary, semi-permanent, and permanent larval habitats were typically characterized by ponding duration of 15–90 days, 90–180 days, and 180 days and above, respectively. The baseline scenario is represented in blue and the irrigation scenario is represented in orange. The darker orange bin is a result of the two overlapping. (**b**) Shows the comparison of the fractional area occupied by non-habitats (less than 15 days) as well as potential temporary, semi-permanent, and permanent larval habitats inside the farms. Each grid cell within the farm was categorized based on its maximum ponding duration.

In the previous section, we showed that irrigation did not have a significant impact on areas outside the farms. Here, we evaluated the stability of the potential larval habitats specifically for the areas inside farms based on the distribution of the maximum duration of ponding for each grid cell within the year as shown in the histogram (Fig. 7a). The total number of cells corresponding to each bin interval of 15 days was expressed as a fraction of the total number of cells in the area inside farms.

From the baseline scenario, 13.2% of the area was not favorable for larval habitats because the maximum duration of ponding in those areas was less than 15 days. The most common maximum ponding duration was between 150 and 165 days, which accounted for more than 20% of the area. This was followed by 15–30 days and 360 days and above which made up 17.6% and 13.8% of the area respectively. With irrigation, there was a general increase in the maximum ponding durations. The most common maximum ponding duration was 360 days and above, accounting for 18% of the area. Noticeably, the area with maximum ponding duration between 210–225 days increased fourfold to 10%. The remaining increase was for 285 days and above. Counter-intuitively, the area that was not conducive as larval habitats (i.e. maximum ponding duration less than 15 days) also increased slightly by 0.6%. This was because irrigation raised the overland flowrate in these areas, mostly near streams, and made them unfavorable for breeding.

In Fig. 7b, we grouped the maximum ponding durations into stability periods corresponding to temporary (2 weeks to 3 months), semi-permanent (3–6 months), and permanent (6 months and above) habitats based on field observations from a study at the site^35^. Temporary habitats such as puddles retain water for a short period while permanent habitats such as stream edges and swamps hold water much longer and are more stable. For the baseline scenario, semi-permanent habitats were the most common, occupying 33.1% of the area, while permanent and temporary habitats also accounted for 29.6% and 24.1% of the area respectively. After irrigation, there was a significant shift from semi-permanent habitats, which reduced to 22.9% of the area, to permanent habitats which increased to 41% of the area. There was also a slight reduction in the extent of temporary habitats to 22.4% of the area.

### Temporal pattern of potential larval habitats

To shed light on the temporal patterns, we evaluated *F*(*WI* > *T*)*,* the fractional coverage of potential larval habitats inside farm, for each day throughout the year. We only focused on the area inside farms since irrigation does not have a significant impact on the area outside farms. As shown in Eq. (3), *F*(*WI* > *T*) is defined as the ratio of *C*(*WI* > *T*), the number of cells for which the *WI* (i.e. persistence of ponding) exceeded *T* days, to *C*_*farm*_, the number of cells within the farm area. *T* is set as 10 days and 15 days, corresponding to critical and normal conditions respectively.

3 $$F\left( {WI > T} \right) = \frac{{C\left( { WI\left( {x,y,t} \right) \ge T} \right)}}{{C_{farm} }},\,T \in \left\{ {10,15} \right\}$$

**Figure 8:**
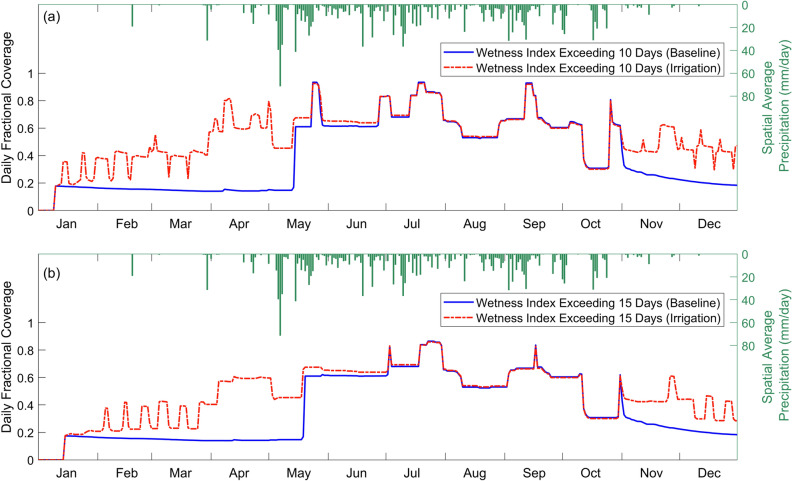
Daily variations in the extent of the potential larval habitats for the year. Time series of the fractional coverage of areas with Wetness Index (*WI*) exceeding (**a**) 10 days and (**b**) 15 days.

In Fig. 8a, F(*WI* > 10) increased steeply on January 10 as *WI* started increasing from 0 at the beginning of the year. For the baseline scenario, the fractional coverage decreased minimally from 0.18 throughout the dry season despite the sporadic spike in precipitation. At the onset of the rainy season, the peak rainfall event of the year from May 5th to May 11th caused a sharp increase in *F*(*WI* > 10) from 0.15 to 0.61 and thereafter, the relentless rainfall maintained the fractional coverage at about 0.6. Throughout the rainy season, there were four recurring peaks at a frequency of about 2 months. Post-rainy season, *F*(*WI* > 10) dropped gradually to below 0.2 after the last peak at the end of October.

For the irrigation scenario, *F*(*WI* > 10) increased during the dry season from January to March with visible cyclical variations between 0.2 and 0.4 due to the rotation of irrigation among the four farms. Subsequently, the spike in rainfall at the end of March combined with the higher antecedent soil moisture from irrigation brought forward the step increase in the fractional coverage to April from May in the baseline scenario. As irrigation stopped at the end of April, *F*(*WI* > 10) gradually dropped back to the same level as the baseline scenario at the end of June. In the dry season from November to December, the fractional coverage started to deviate from the baseline scenario again with cyclical fluctuations, gradually decreasing towards the end of the year.

In Fig. 8b, F(*WI* > 15) remained largely the same for the dry season but the peaks were moderated in the rainy season, compared to *F*(*WI* > 10). There was one less peak at the end of May in the early rainy season because the watershed did not accumulate enough rainfall for the persistence of the ponded areas to exceed 15 days. Specifically, for the irrigation scenario, the increase in fractional coverage during the dry season was moderated and less sensitive to the spikes in rainfall. Similarly, irrigation resulted in the early onset of the steep increase in *F*(*WI* > 15) in April following the spike in rainfall at the end of March. Also, it took two months after the end of irrigation in April for the fractional coverage to return to the same level as the baseline.

**Figure 9:**
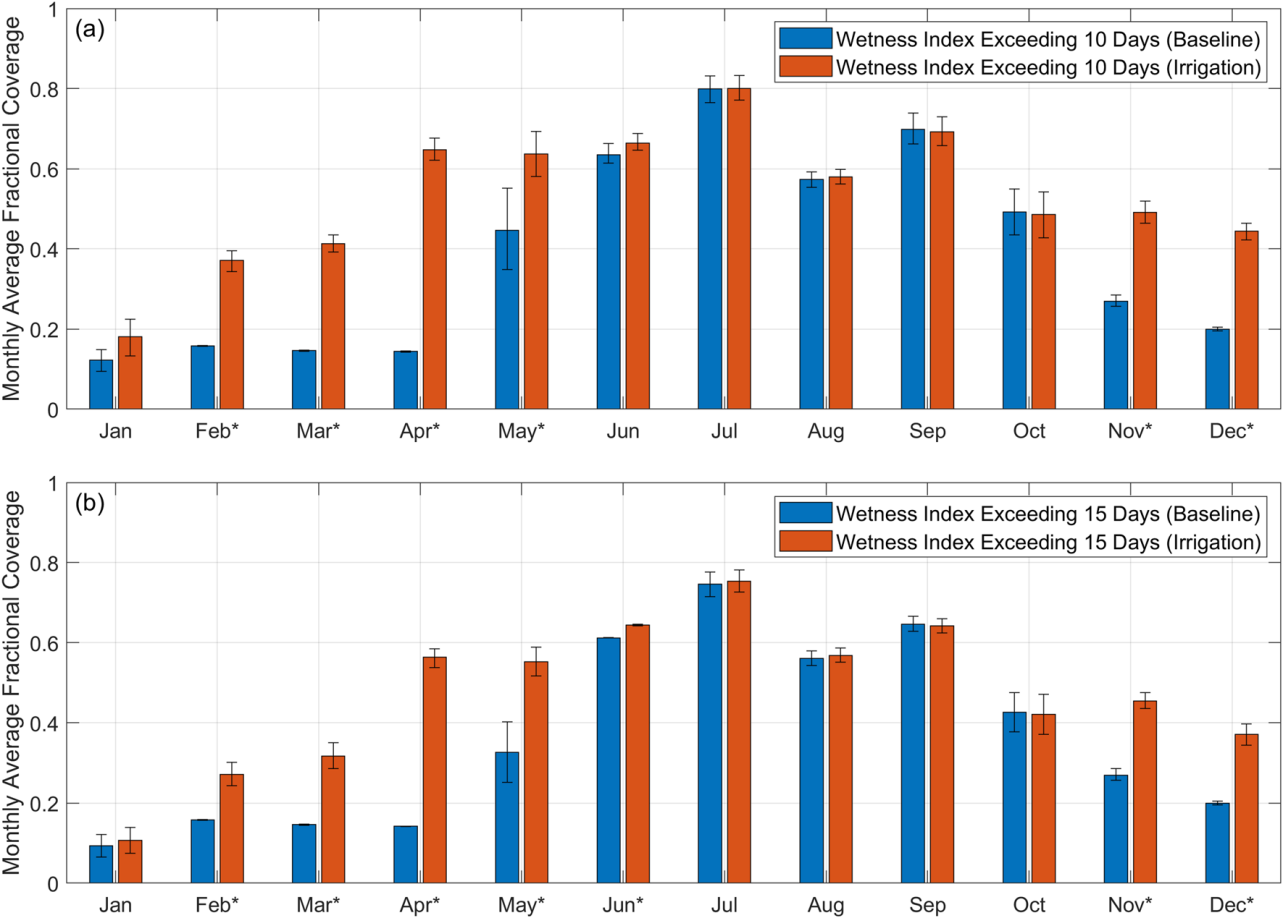
Monthly variation in the extent of the potential larval habitats for the year. Monthly mean fractional coverage of areas with a probability of *WI* exceeding 10 days (**a**) and 15 days (**b**). The 95% confidence interval is indicated at the top of each bar chart. The asterisks (*) next to the month on the x-axis indicate that irrigation increased the fractional coverage of the potential larval habitats for the month from the baseline scenario based on a 2-sample t-test (*p* < 0.01).

From *F*(*WI* > 10) and *F*(*WI* > 15), we calculated the corresponding monthly mean, *MF*(*WI* > 10)*,* and *MF*(*WI* > 15) as well as the 95th confidence interval as shown in Fig. 9. In Fig. 9a, *MF*(*WI* > 10) in the baseline was the highest for the months between June and September, constituting a four-month window in which at least 50% of the area was conducive for larval habitat formation. Of the four months, the highest monthly mean fractional coverage was in July at 79.9%. Irrigation extended the window to include the months of April and May. The monthly mean fractional coverage increased 4.5 times to 64.3% in April and 1.4 times to 63.7% in May. The *MF*(*WI* > 10) for the rest of the months in the window (i.e. June to September) remained one of the highest but the increase due to irrigation was not statistically significant (*p* > 0.01). July remained as the month with the highest monthly mean fractional coverage at 80.0%. In Fig. 9b, *MF*(*WI* > 15) was generally slightly lower than *MF*(*WI* > 10) for both the baseline and irrigation scenarios but the general trends were the same.

## Discussion

### Impact of irrigation on spatiotemporal distribution of potential larval habitats

This study, for the first time, employed an integrated hydrological model to predict the proliferation of potential larval habitats around an irrigated field during the dry and rainy seasons. Irrigation resulted in a much higher probability to find potential larval habitats in the dry season. Although irrigation was not applied during the rainy season, a slight increase in the probability of potential larval habitat occurrence was also observed due to the higher antecedent soil moisture from the dry season promoting the formation of larval habitats further. Considering that larval habitat availability is one of the direct predictors for vector abundance, our findings tie in with local studies^35,50,51^ in the Arjo-Didessa sugarcane plantation which showed a higher occurrence of *Anopheline* mosquito larval habitats, larval productivity and abundance in the irrigated areas than non-irrigated areas in both dry and wet seasons.

Separately, previous studies have identified irrigation schemes as the cause for prolonging mosquito season and extending the period of malaria transmission^52,53^. In our study, it was found that the stability of potential larval habitats was similarly prolonged, with a significant shift from semi-permanent (3–6 months) to permanent habitats (6 months and above) in the irrigated farm areas. Originally, the semi-permanent habitats were the most common, occupying a third of the area inside farm without irrigation. In the irrigation scenario, the area of the permanent habitats became the most common, increasing to more than 40% of the area inside farm.

The temporal variations in the extent of the potential larval habitats indicate that rainfall exerts a strong influence. It is common that peak mosquito breeding seasons follow rainfall in the tropics^54^. In our study, the occurrence of potential larval habitats was highest for the period from June to September, during which at least 50% of the area inside farms were potential larval habitats. This overlaps with a significant portion of the rainy season. From the irrigation scenario, we observed that irrigation dampened the seasonality of the potential larval habitats by increasing the wetness index in the dry season and extending the peak larval habitat occurrence window to include the months of April and May. Elsewhere in Africa, irrigation is also known to reduce the dependence of larval habitat patterns on rainfall, changing them from seasonal to perennial^55^.

This calls for the need to modify current irrigation strategies and develop tailor-made interventions to mitigate mosquito breeding around irrigated fields in order to combat malaria. Ideally, the aim is to optimize irrigation to minimize larval habitat availability while meeting crop water requirement. To this end, our model has the flexibility to simulate different types of irrigation such as flood irrigation, groundwater irrigation, sprinkler irrigation and drip irrigation as well as water allocation strategies. Hence, beyond predicting potential larval habitats, the model can help configure the outline of the irrigation design and sieve out some of the more pertinent and effective strategies. For example, the main method of irrigation in Ethiopia is surface irrigation which has low implementation cost but is known to be inefficient in water use^56^ and can aggravate malaria transmission by providing an ideal larval habitat^57^. For regions where resources are limited, our model can be used to identify irrigated farms with the most serious ponding to prioritize the installation of higher water efficiency but also costlier irrigation systems such as drip or sprinkler irrigation. It can also be coupled with a water allocation algorithm^58^ to investigate the larval habitat distribution under more complex water management operations.

### The implication of model assumptions and simplifications on results

In this study, we chose to simulate the surface layer soil saturation at 50 m resolution, coupled with a threshold, to quantify ponding instead of explicitly simulating the surface water depth. Without high resolution and accurate topographic information, it was not feasible to achieve the latter at the scale of the larval habitats surveyed in the study area, most of which measure less than 100 m^2^ each. It has been shown that a minimum of 3 model grid cells is required across the width of the land depression for a good balance between accuracy and computational effort when simulating a flood extent^59^. This requires accurate data with a minimum resolution of 3 m that can only be obtained using Lidar, RTK (Real-Time Kinematics), or PPK (Post Processed Kinematics) with aircraft or drone, which is time consuming and expensive. Regardless, the computational efficiency of the model with high-resolution DEM will pose another challenge even if they were available. As such, the model is not intended to pinpoint the exact location of each larval habitat in the study area. Instead, it provides information on the overall likelihood of ponding for each grid cell based on the interactions between system properties and forcing variables at various temporal and spatial scales. The strength of this approach is that the model can afford to run on a regional scale at a fairly high spatial resolution while keeping computational requirements manageable. Furthermore, all the primary data used are freely available for all regions of the world and hence, this framework provides a great opportunity to extend potential larval habitat simulation into other locations without incurring high data acquisition cost.

In terms of performance, the model was able to predict ponding at all the validation points after calibration. As shown in Supplementary Fig S6, the probability of detection for both calibration and validation reached 1 (i.e. all the points are detected) at the optimal soil saturation threshold *θ* of 0.48. However, this does not mean the model is perfect as the calibration and validation did not account for overprediction at locations without ponding since the survey was only for locations with ponding. This will be resolved in future by ongoing field survey efforts toward compiling a more spatiotemporally comprehensive dataset including locations without ponding. Another limitation was that calibration was only performed on the soil saturation threshold but not the ParFlow model per se although the simulated soil saturation was realistic from its general behavior. This was due to the lack of data to verify surface and subsurface flow rates at a relevant spatial scale. With the collection of more data in future, the key parameters can be fine-tuned to improve model predictions. A more detailed irrigation schedule would also provide insight into the water usage, irrigation management, and surface land cover during sugarcane growth. Such data could serve as a better guide in modeling pond formation that incorporates the effect of irrigation. Unfortunately, records for canal water flow, water usage, and field operations were not available at the time of visit to the Arjo-Didessa Sugar Factory, and only the summary data and annual plan could be obtained.

## Conclusion and future prospects

Using high resolution distributed hydrologic modeling with remotely sensed data, we demonstrated a quantitative assessment of potential malaria vector larval habitats in terms of the spatial distribution and temporal variation. We also evaluated the relative influence of key environmental processes such as rainfall and irrigation on the habitats. Results indicated a higher probability to find potential larval habitats inside the farms, at around 40% of the year, than outside the farms, at less than 10% of the year. Our model also showed that rainfall exerted a strong influence on larval habitat availability based on predictions that at least 50% of the area inside farms were potential larval habitats from June to September during the rainy season. Further, modeling revealed that irrigation increased the probability of finding potential larval habitats inside the farms to 67%. Irrigation also dampened the seasonality of the potential larval habitats such that the peak larval habitat occurrence window during the rainy season was extended. Lastly, the stability of larval habitats was prolonged, with a significant shift from semi-permanent habitats to permanent habitats lasting beyond 6 months, pointing to the impact of irrigation in creating conducive mosquito habitats throughout most of the year.

Since the effectiveness of major malaria vector control measures is decreasing due to mosquito insecticide resistance and outdoor transmission, the role of LSM as a supplementary vector control tool to reduce malaria transmission becomes more significant. As such, hydrologic modeling with publicly available data, presented herein, constitutes a promising direction in terms of providing a dynamic and systematic approach for the identification and elimination of larval habitats by environmental modification and manipulation. For hydrologic modeling to fulfill its promise in the area, enhanced observational efforts are required in future. Thorough calibration and validation will be critical in evaluating the robustness and quantifying the uncertainty of the model.

Food security will bring economic growth and remains one of the priorities in Africa. To this end, investment in dams and irrigation systems is increasing rapidly in Africa over the past decade. Unfortunately, this might increase the risk of malaria due to environmental modifications and microclimate changes. The broader goal of our research is to harness the hydrological results, along with other epidemiological, entomological and social-economic factors, to translate the knowledge of potential larval habitats to useful information on the spatio-temporal distribution of malaria transmission risks. Remotely sensed data can enable this type of modeling in data-scarce regions where malaria presents a grave threat. This framework has great potential to integrate with malaria epidemiologic modeling such as EMOD^60^ to predict malaria risk under different environmental modifications to guide decision-making in water resource management, changes to agricultural practice, and disease prevention.

## Supplementary Information

Supplementary Information 1.

Supplementary Video S1. Animation of Baseline Scenario in 2018 with Precipitation. An animation of the simulated soil saturation dynamics for the baseline scenario with daily precipitation during Year 2018 in the study site.

Supplementary Video S2. Animation of Irrigation Scenario in 2018 with Precipitation. An animation of the simulated soil saturation dynamics for the irrigation scenario with daily precipitation during Year 2018 in the study site.
