# Supplementary material for: Predicting distribution of malaria vector larval habitats in Ethiopia by integrating distributed hydrologic modeling with remotely sensed data
Source: Sci Rep. 2021 May 12;11:10150. doi: 10.1038/s41598-021-89576-8 (PMC8115507; doi:10.1038/s41598-021-89576-8)
Supplement: Supplementary file 1 — Supplementary Information 1. [file 41598_2021_89576_MOESM1_ESM.pdf]

Supplementary Information

Predicting Distribution of Malaria Vector Larval Habitats in Ethiopia by Integrating Distributed Hydrologic Modeling with Remotely Sensed Data

Ai-Ling Jiang[1,*], Ming-Chieh Lee[2], Guofa Zhou[2], Daibin Zhong[2], Dawit Hawaria[3,4], Solomon Kibret[2], Delenasaw Yewhalaw[4,5], Brett F. Sanders[1,6], Guiyun Yan[2,*], Kuolin Hsu[1,*]

1. Department of Civil and Environmental Engineering, University of California Irvine, Irvine, California, USA
2. Program in Public Health, University of California Irvine, Irvine, California, USA
3. Yirgalem Hospital Medical College, Yirgalem, Ethiopia
4. Tropical and Infectious Diseases Research Center (TIDRC), Jimma University, Jimma, Ethiopia
5. Department of Medical Laboratory Sciences, Institute of Health, Jimma University, Jimma, Ethiopia
6. Department of Urban Planning and Public Policy, University of California Irvine, Irvine, California, USA

* jiangal@uci.edu

* guiyuny@uci.edu

* kuolinh@uci.edu

This file contains the Supplementary Note, Supplementary Table S1 and Supplementary Figure S1 to Figure S6.

Supplementary Note: Additional Information for Materials and Methods

1. Input Data.

Using GRASS 7.6, the 1 arc-second digital elevation model (DEM) from SRTM was resampled to 50 m grid resolution. Subsequently, the resampled DEM data was hydro-conditioned to ensure that the drainage networks are connected using the global slope enforcement approach[1] and converted to ground surface slopes as an input to ParFlow. The land cover type data used in CLM was determined by 30 m resolution Landsat-8 data, which was first filtered by cloud cover and processed with an unsupervised classification approach using k-mean clustering into ten classes in ENVI. The ten classes were resampled to 50 m resolution and then grouped into the International Geosphere-Biosphere Programme (IGBP) land cover types and checked against Google Earth images for consistency. The dominant IGBP types for the study area are cropland and grassland.

To characterize the subsurface, the soil taxonomy distribution (Supplementary Fig S2) for the top 2 m from the surface was referenced from the SoilGrids250m TAXOUSDA dataset[2] and ranked by their relative permeability. Each soil type was then assigned a saturated hydraulic conductivity within the range of 0.0015-0.015 m/h[3] characteristic of either clay or clay loam. The saturated hydraulic conductivity of the deeper zone beyond the top 2 m was assigned an averaged value of 0.11 m/h based on GLHYMPS 2.0. The depth to bedrock data from SoilGrids250m BDRICM dataset[2] was used to delineate the bedrock zone and the hydraulic conductivity was set to 0.00001 m/h to render the corresponding grid cells impermeable.

For the climate forcing, 0.04 degree by 0.04-degree precipitation data was resampled to the model grid using bilinear interpolation while the rest of the forcing data fields were relatively coarser at

either 0.25 degree by 0.25 degree or 0.5 degrees by 0.625 degrees so an average value was used for the entire domain. All the forcing data were pre-processed in NCAR Command Language (NCL) and input to the model hourly.

The list of model input data can be found in Supplementary Table S1.

2. Model Simulation.

In this study, a baseline was generated over the year of 2018 followed by a scenario with the implementation of a synthetic irrigation scheme during the dry season. The watershed model spans an area of 208 km^2 and has a high resolution, both spatially and temporally. The model resolution is 50m and there are 332 by 248 cells. The model was divided into 10 subsurface layers and the thickness of each layer varies, decreasing gradually towards the surface. For the irrigation scenario, the sugarcane plantation in the study area was grouped into four farms. The soil textural classes of the study area are clay and heavy clay soils and the peak admissible infiltration rate of these soils was 4mm/hr. The efficiency of sprinkler irrigation was estimated at 75%. Hence, a 10 mm/day irrigation operational module using the hose-moving sprinkler system was applied on alternate farms. The daily irrigation time was 22 hours per day and the irrigation interval of 10 days proposed by sugar factory agronomists was adopted.

The model was initialized in two stages. In the first stage, the CLM component was not activated and the initial water table was set at the surface without meteorological forcing. The simulation was run until the average water table reached 2 m below the land surface. During this process, the water drains from the mountainous areas to valleys, forming rivers and streams naturally. Next, the CLM component was activated and the transient meteorological forcing from the year of 2017 was repeated three years for the model to reach a sufficient state of equilibrium.

All model simulations were carried out on the High-Performance Computing Cluster at the University of California, Irvine. A 1-year simulation required 88 CPU cores and about 12 days to complete.

Supplementary Figure S4 shows the time series of precipitation, temperature, and simulated surface layers soil saturation for the baseline simulation and streamflow at points A, B, and C (see Fig 1). Supplementary Video S1 and S2 demonstrate the simulated soil saturation dynamics for the baseline condition and irrigation scenario with the daily precipitation of the Year 2018 in the study site.

Supplementary Figure S5 compares the time series of the simulated surface layers soil saturation for the baseline and irrigiation simulations. In general, the surface layer soil saturation fluctuated in tandem with precipitation. Irrigation during the dry season increased the surface layer soil saturation and the pattern of fluctuation was influenced heavily by the alternating irrigation scheme. Also, the surface layer soil saturation varied depending on the location. For instance, Point B which is in Farm 2 had the highest soil saturation and was near saturation point most of the time from August to September because it is located close to a small stream. The baseline results show that the surface soil saturation of the four locations was relatively low in the dry months (January to April), ranging from 5% to 15% but could go up to as high as 100% in the rainy season (May to October). In the scenario with irrigation, the soil saturation increased two-fold to about 50 in the dry months. This contributes to surface water detention, which can potentially generate more breeding sites. As the irrigation ended in April with the onset of the rainy season, the increase in the saturation of the irrigation scenario over the baseline gradually reduced until they were both the same after more than a month.

3. Soil Saturation Threshold θ Parameterization

3.1 Aquatic Habitat Survey

The aquatic habitats were surveyed during the dry (December 2017–February 2018) and rainy (June 2018–August 2018) seasons[4]. Mosquito larvae were sampled following the WHO standard larval survey procedure using a standard dipper (350 ml). Larvae were identified morphologically and sorted by genus as *Anopheles* and *Culex* in the field. Environmental variables such as habitat type, habitat dimension and land cover type were recorded using the Android-based tablet PC with ODK Collect application[5] while the coordinates of each surveyed aquatic habitat were captured with the built-in Geographic Positioning System (GPS) sensor. All the data were eventually uploaded onto the ODK Aggregate Server[5] on the cloud-based Amazon Web Services (AWS).

Post survey, all the aquatic habitats and larvae survey metadata were pulled out from the ODK Aggregate[5] MySQL database (version 5.7, Oracle Corporation, California, USA) and analyzed with JMP (version 14, SAS Institute, North Carolina, USA) and Microsoft Excel (Version 2019, Microsoft Corporation, Washington, USA). Spatial data aggregation, analysis, and visualization were produced with ArcGIS Pro 2.5[6] The locations of the surveyed aquatic habitats are shown in Fig 3.

3.2 Calibration and Validation

The soil saturation threshold θ for the rainy season from May to October of the baseline scenario was calibrated using the survey data in the same period to minimize the influence of dry season irrigation on the parameterization. This is because irrigation was not accounted for in the baseline scenario and was only approximated by a simplified scheme in the irrigation scenario. Of the 134 samples for the year 2018, some of the surveyed aquatic habitats such as man-made ponds, tire

track puddles, and animal footprints which could not be simulated by the hydrologic model were omitted, leaving 102 samples for calibration and validation as shown in Fig. 3.

The objective of the calibration was to maximize the probability of detection (*POD*), which determines if the model can predict an aquatic habitat successfully. Other measures which can capture overprediction were not chosen here as the field data only cover locations with ponding and it is challenging to definitively rule out small puddles within the grid cell

using satellite data. As shown in Equation (S1), the *POD* was calculated based on the ratio of the number of successful predictions or hits, *H*, to the total number of samples, *S*. In a successful prediction, the *WI* is at least 1 day given that an aquatic habitat is sampled at the same time and location. The *POD* ranges from 0 to 1, with 1 corresponding to perfect detection.

$$POD = \frac{H}{S} \tag{S1}$$

As the same dataset was used for both calibration and validation, 75% of the samples were randomly selected for the former and 25% for the latter. To ensure the relevance of the calibrated saturation threshold, bootstrapping was performed by randomly resampling the same dataset based on the 75:25 ratio with replacement to derive 1000 combinations as a representation of all the possible combinations. Each combination was used to calculate the *POD* corresponding to the saturation threshold within a predefined range of 0.4 and 1, at intervals of 0.02. To determine and assess the reliability of the optimal threshold, the average *POD*, and the associated 95% confidence interval values were computed for each saturation threshold.

Supplementary Figure S6 shows the results from the calibration and validation of the bootstrap samples. It can be observed that the average *POD* of the validation was consistent with that of the calibration for each saturation threshold. The confidence interval for validation was wider than

that of calibration due to the smaller number of survey samples used. The average *POD* for both the calibration and validation samples first reached 1 when the saturation threshold value decreased to 0.48. At the same time, the confidence intervals decreased to close to zero, indicating high reliability. Hence, the optimal saturation threshold was set as 0.48 to ensure that all the observed aquatic habitats were captured in the model.

Supplementary Figures

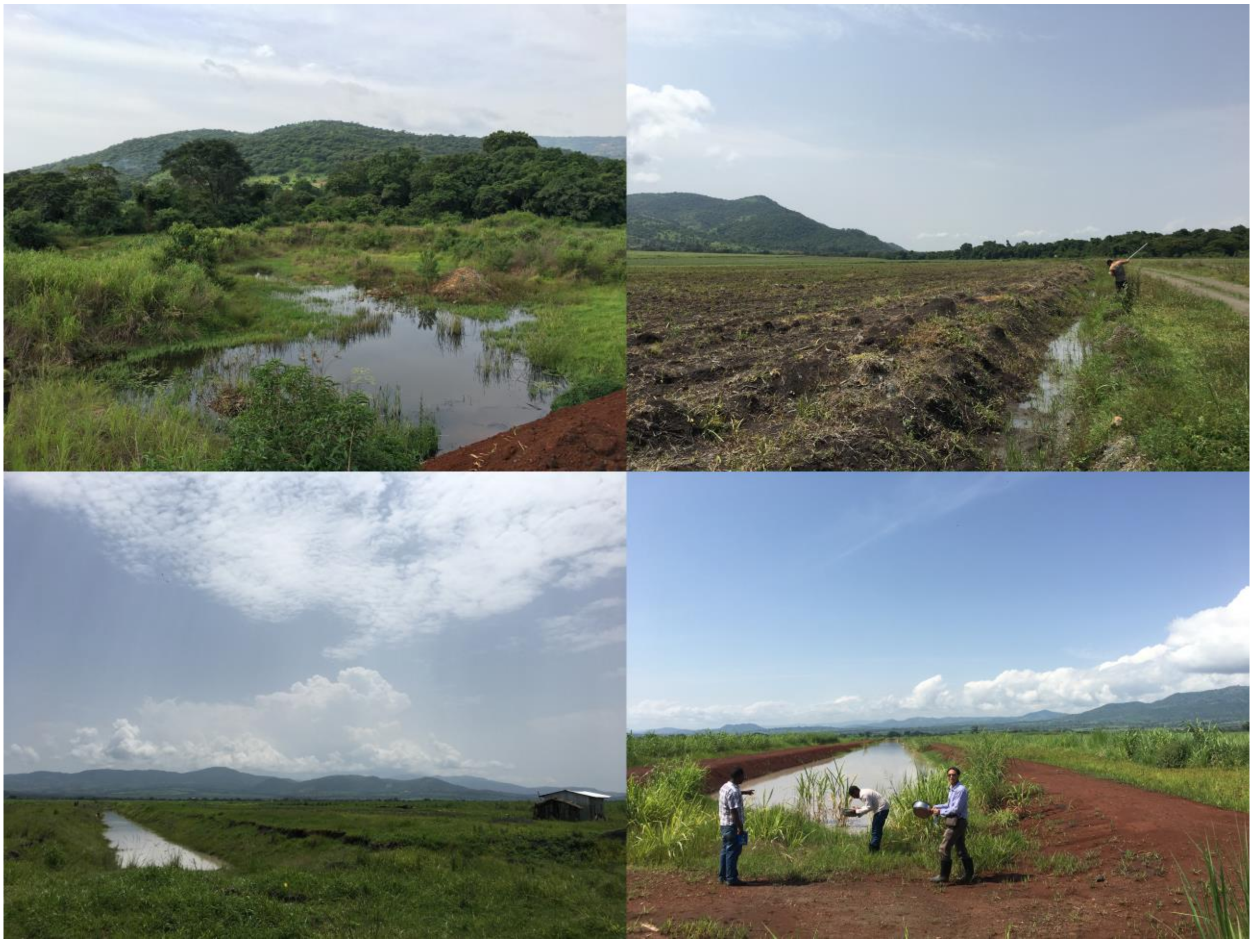

Supplementary Figure S1. Typical rain-fed ponding in the study area in Arjo. The study site was in the valley bottom which is characterized by clay and clay loam with low permeability soil with high annual precipitation around 1500 mm which mainly distributed in the rainy season from May to October. This rainfall distribution and irrigation during the dry season created many long-term surface water storages that provide potential mosquito habitats.

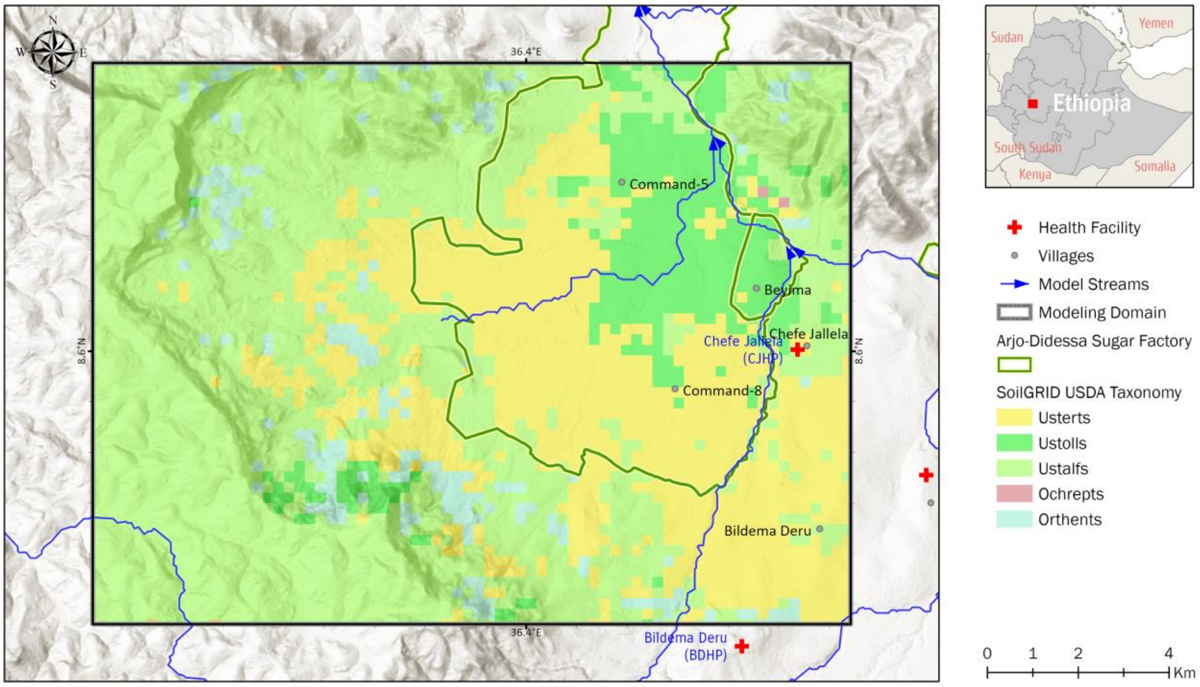

Supplementary Figure S2. The distribution of the top 2mm soil types in USDA soil taxonomy from SoilGrids250m TAXOUSDA dataset. Most soil types in this area are characterized as clay or clay loam with low permeability ranging from 0.0015 to 0.015 m/h.

(a)	1st Year												2nd Year												1st Ratoon			
Mn	1	2	3	4	5	6	7	8	9	10	11	12	1	2	3	4	5	6	7	8	9	10	11	12	1	2	3	4
Sym.	MA	H	H	LW	P	P	P	P	RF	RF	IR	IR	IR	IR	IR	IR	RF	RF	RF	RF	RF	RF	MA	MA	MA	H	IR	IR

(b)	1st Ratoon												2nd Ratoon												Virgin Planting			
Mn	1	2	3	4	5	6	7	8	9	10	11	12	1	2	3	4	5	6	7	8	9	10	11	12	1	2	3	4
Sym.	MA	H	IR	IR	RF	RF	RF	RF	RF	RF	MA	MA	MA	H	IR	IR	RF	RF	RF	RF	RF	RF	MA	MA	MA	H	H	LW

Supplementary Figure S3. Arjo-Didessa Sugar Factory sugarcane plantation irrigation schedule. A normal sugar planting schedule (a) includes a 2-year cycle for virgin and (b) 1-year cycle for following (2~8) ratoons. The symbol for each stage: **MA**: Maturity/water withdraw; **H**: Harvesting; **LW**: Land work; **P**: Planting; **RF**: Rainfed; **IR**: Irrigation.

[illegible]

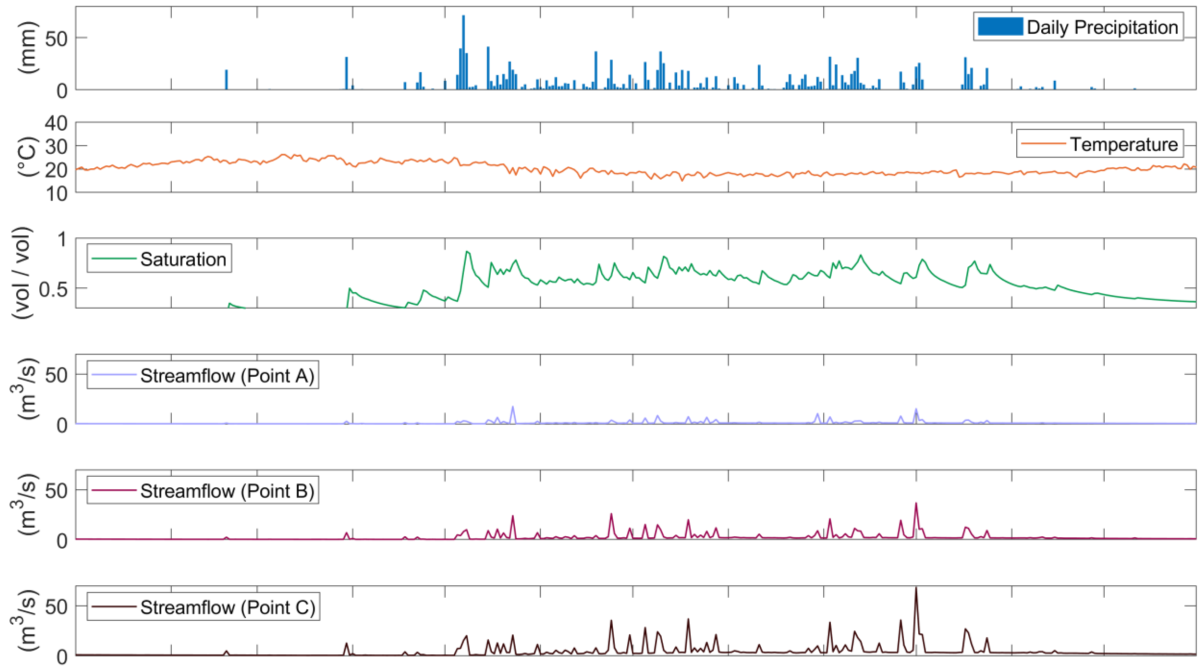

Supplementary Fig

layers soil saturation for the baseline simulation and streamflow at point A, B, and C (see

Error! Reference source not found.

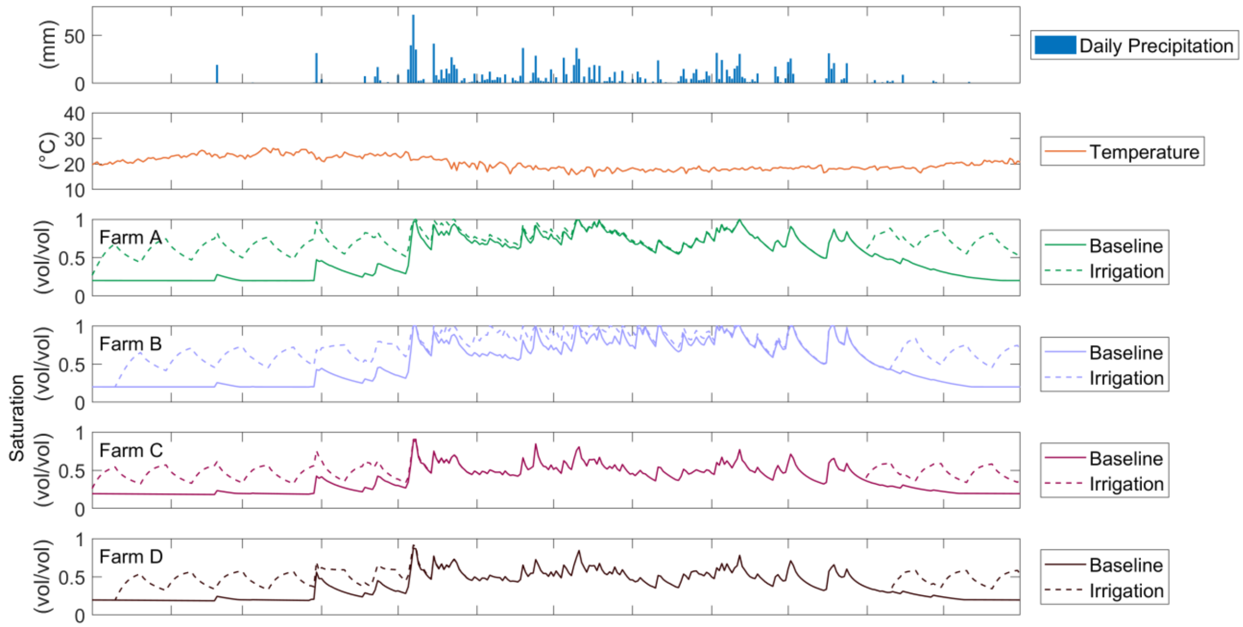

Supplementary Figure S5. Time series of precipitation, temperature, and simulated surface layers soil saturation for the irrigation scenario at a specific point of each of the four farms (see Fig 1). The precipitation and temperature profiles were spatially-averaged. The soil saturation profile reflects the 2-cycle rotational irrigation schedule whereby Farm #1 and Farm #3 were irrigated first followed by Farm #2 and Farm #4.

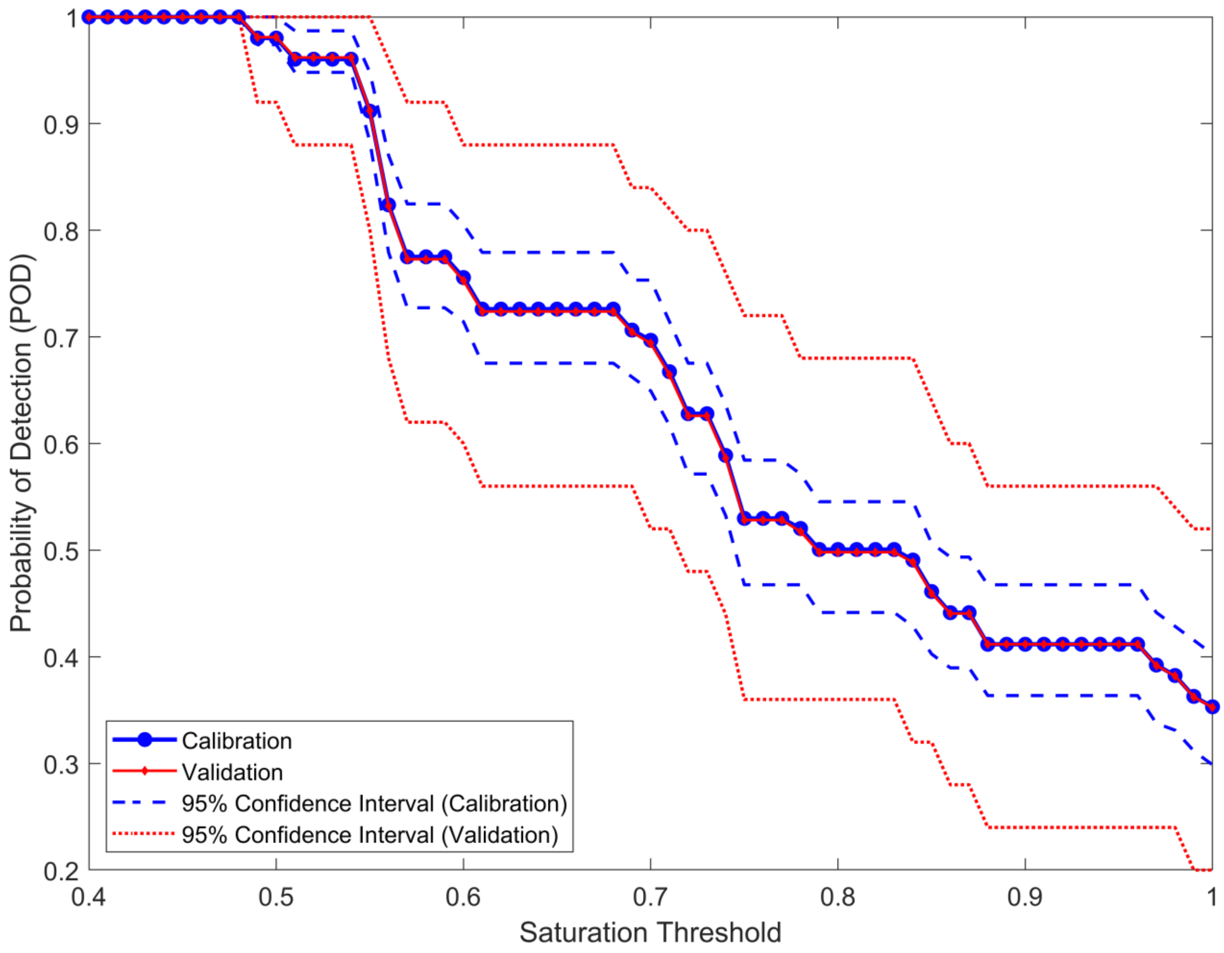

Supplementary Figure S6. Model performance from the calibration and validation of the saturation threshold. The 75% of the samples were selected for calibration and the other 25% for validation and the process was repeated 1000 times. The mean POD values from the calibration and validation are represented by the blue line with a circle marker and red line with a cross marker, respectively. The corresponding 95% confidence intervals are indicated by the dotted blue and red lines, respectively.

Supplementary Tables

Supplementary Table S1. ParFlow-CLM model input data and their properties

Variable	Resolution	Input Type	Latency	Source
**Topography**	30-meter	Distributed	-	NASA Shuttle Radar Topography Mission, (SRTM) Version 3.0[7]
**Precipitation**	0.04°×0.04°, 1-hourly	Distributed	~1 hour	Precipitation Estimation from Remotely Sensed Information using Artificial Neural Networks-Cloud Classification System, (PERSIANN-CCS)[8,9]
**Short-Wave radiation**	0.25°×0.25°, 3-hourly	Distributed	4.5-5.5 month	Global Land Data Assimilation System, (GLDAS)[10]
**Long-Wave radiation**	0.25°×0.25°, 3-hourly	Distributed	4.5-5.5 month	GLDAS
**Air Temperature**	0.25°×0.25°, 3-hourly	Distributed	4.5-5.5 month	GLDAS
**Atmospheric Pressure**	0.25°×0.25°, 3-hourly	Distributed	4.5-5.5 month	GLDAS
**Water-vapor specific humidity**	0.25°×0.25°, 3-hourly	Distributed	4.5-5.5 month	GLDAS

**North-to-South Component of Wind Speed**	0.5°× 0.625°, 1-hourly	Distributed	4.5-5.5 month	The second version of Modern-Era Retrospective analysis for Research and Applications, (MERRA-2)[11]
**East-to-West Component of Wind Speed**	0.5°× 0.625°, 1-hourly	Distributed	4.5-5.5 month	MERRA-2[11]
**Land use**	30-meter 16-day	Distributed	~12 hours	Landsat 8[12]
**Soil type**	250-meter	Distributed	-	SoilGrids250m, TAXOUSDA[2]
**Depth to Bedrock**	250-meter	Distributed	-	SoilGrids250m, BDRICM[2]
**Near Surface Permeability (< 100 m)**	Regional Scale	Distributed	-	GLobal HYdrogeology MaPS 2.0 (GLHYMPS, 2.0)[13]

Supplementary References

1. Barnes, M. L., Welty, C. & Miller, A. J. Global Topographic Slope Enforcement to Ensure Connectivity and Drainage in an Urban Terrain. *J. Hydrol. Eng.* **21**, (2016).

2. Hengl, T. *et al.* SoilGrids250m: Global gridded soil information based on machine learning. *PLoS One* **12**, e0169748 (2017).

3. Soil Science Division Staff. Soil Survey Manual, USDA Handbook 18. 145 (2017). doi:10.2307/1233734

4. Hawaria, D. *et al.* Effects of environmental modification on the diversity and positivity of anopheline mosquito aquatic habitats at Arjo-Dedessa irrigation development site, Southwest Ethiopia. *Infect. Dis. Poverty* **9**, (2020).

5. Hartung, C. *et al.* Open Data Kit: Tools to Build Information Services for Developing Regions. in *Proceedings of the 4th ACM/IEEE International Conference on Information and Communication Technologies and Development* (Association for Computing Machinery, 2010). doi:10.1145/2369220.2369236

6. ESRI. ArcGIS Pro. (2020).

7. JPL, N. NASA Shuttle Radar Topography Mission Global 1 arc second. (2013). doi:https:doi.org/10.5066/F7PR7TFT

8. Hong, Y., Hsu, K.-L., Sorooshian, S. & Gao, X. Precipitation estimation from remotely sensed imagery using an artificial neural network cloud classification system. *J. Appl. Meteorol.* **43**, 1834–1853 (2004).

9. Nguyen, P. *et al.* The CHRS data portal, an easily accessible public repository for PERSIANN global satellite precipitation data. *Sci. Data* **6**, 1–10 (2019).

10. Rodell, M. Basin scale estimates of evapotranspiration using GRACE and other observations. *Geophys. Res. Lett.* **31**, L20504 (2004).

11. Gelaro, R. *et al.* The modern-era retrospective analysis for research and applications, version 2 (MERRA-2). *J. Clim.* **30**, 5419–5454 (2017).

12. Center, U. S. G. S. (USGS) E. R. O. and S. (EROS). LANDSAT 8 OLI/TIRS Collection 1. (2013). doi:https:doi.org/10.5066/F71835S6

13. Gleeson, T., Moosdorf, N., Hartmann, J. & van Beek, L. P. H. A glimpse beneath earth's surface: GLobal HYdrogeology MaPS (GLHYMPS) of permeability and porosity. *Geophys. Res. Lett.* **41**, 3891–3898 (2014).
